# The Study of Dental Surgery and the Means Thereto

**Published:** 1881-09

**Authors:** John Tomes


###  THE DENTAL REGISTER.

VOL. XXXV.] SEPTEMBER, 1881. [No. 9.

### THE STUDY OF DENTAL SURGERY, AND THE MEANS THERETO.

*Abstract of a Paper read before Section XII. of the International Medical Congress, Aug. 5th,* 1881.

BY JOHN TOMES, F.R.S., L.D.S.E., &C., M.R.C.S.ENG.

Dental Surgery has within the present century, by the full consent both of the medical and general public, developed into a well-defined speciality. The medical practitioner refers all dental cases to a dentist, where one is at hand, and the general public select him as the fittest to help them in all cases of dental trouble. No apology, therefore, need be offered for the separate practice of Dental Surgery, neither need arguments be put forward in support of its continuance as a distinct branch of surgical practice. The necessities of society on the one hand and the technical requirements of the dentist on the other hand, have determined the condition of separateness. But this great international meeting affords a fitting occasion to inquire how the accepted condition can for the future be met, so that the public may be best served, for therein rests the sole cause for our presence, either as special or indeed as any kind of practitioners whatever.

Utility alone is the excuse for the dentist's existence, and the full recognition of this fact brings us to the question of how and by what available means he can become most useful? How he can best fulfill the trust imposed on him as a specialist, bearing in mind that on account of his supposed superior special knowledge, he is consulted, and thus assents to the belief that the dentist is far more capable than the general surgeon in the treatment of dental ailments. Clearly his honour—nay, even his integrity—is pledged to render himself in the highest degree capable of discharging to the fullest the freely-accepted duties. In admitting the social necessity for the presence of the special practitioner, the need for his special education is conceded, and it is to the wide question of what should be the education of the dental practitioner, for the determination and the development of which, we, as practitioners and teachers, are responsible, that I would call the attention of the meeting.

Before proceeding further, however, let me state that I wish it to be understood that all I have to say upon the subject of dental education, applies only to those who have yet to be educated, and to those who possess neither unusual fitness nor unfitness for the pursuit of dental studies. And furthermore I desire to state that any opinions I may express as to what can and should be done, are intended to apply only to education in England. It will be for the representatives of other nationalities, to tell us what system of education is most applicable and suitable in their respective countries.

In the first and second decades of the present century, dental practitioners were few in number, and for the most part, but not in all cases, members of the medical profession, who at the onset of practice had but a slender knowledge of the duties of the dental surgeon even as they were then understood, or at best they had such an amount of knowledge only as the accident of a good or bad private instructor might impart, in all constructive matters depending from the first upon the assistance of dental mechanists. Other persons commenced their career as young men or boys in the laboratory of a dental practitioner, acquiring therein in the course of an apprenticeship extending over five or even seven years, great manual skill, but whose claim to surgical knowledge at the expiration of pupilage, could not be sustained. Yet from this class of persons some of the most distinguished practitioners of the last generation were derived. The one spent those years, when to learn is easy and authority in the teacher is effective, in the acquisition of manual skill; the other in the acquisition of medical, I will not say surgical, knowledge, in the strict meaning of the term surgical. Hence it was that practice was approached from two wholly different sides, and resulted in the production of practitioners of two distinct classes; one competent to advise, the other competent to treat, but neither fully competent both to say what should be done and to do it effectively.

Towards the end of the second decade dentists began to increase in number, and each year up to the middle of the century brought new candidates for practice, the vast majority of whom came directly from the dental laboratory, and were, for the most part, inferior in general education to the surgeon, in whose medical knowledge they had no share. Out of this educational difference arose an inter-professional division, not to say jealousy, in which society took but little interest, each person selecting for himself a practitioner from whom he hoped to secure all the advantages that treatment could effect, and the choice as often fell upon the unqualified as upon the surgically qualified practitioner. Among the more intelligent practitioners it came to be freely admitted that dental education, from its one-sided character, was in a very unsatisfactory condition; and after some few years of discussion, the opinion was generally accepted that the general and special portion of the dental training should go on simultaneously; so that both manipulative skill and surgical knowledge should be acquired in the days of our youth, when the power to acquire is at its best, and at the only time, indeed, when a high degree of manipulative skill can be acquired.

A consensus of opinion as to requirements having been obtained, effectual action soon followed.

But we were not the first to recognize the necessity of a systematic dental education. Our American brothers had not only felt but provided for the need, in the organization of dental colleges; and we, in following in their footsteps, and profiting by their experience, accepted an obligation which should at all times be freely acknowledged.

The history of the organization of the past and present dental colleges of America has been published in "The History of Dental and Oral Science in America," 1876. From this and from Dr. Eliot's address, delivered before the American Academy of Dental Science, 1879, and from the prospectuses of the American dental colleges, I shall take such facts, as should be stated in acknowledgment of the work of our predecessors, and of those differences of method or requirements in education, which differences of attendant social or national opinions, have rendered desirable or necessary.

In order to arrive at a full understanding of the constitution of the American dental colleges, it will be desirable to refer very briefly to the state of medical education and of medical colleges, upon the lines of which the dental schools were, to a certain extent, of necessity drawn. For this information I am wholly indebted to the "Special Report upon Medical Education and Medical Institutions in the United States of America, 1776-1876," prepared for the United States Bureau of Education by N. S. Davis, A. M., M.D., and to the address on "The Relations of the Medical Profession to the State," by D. B. St. John Rosa, M.D., 1879.

In early days Scotch graduates, settled in America, organized a university drawn after the northern model, but it was soon found that educational demands upon the student, readily met in Scotland, were altogether beyond the powers of the youth of a newly settled country.

Hence, to avoid failure, the standard had to be lowered in favor of private pupilage. After the War of Independence, according to Dr. Davis, universities or colleges sprung up in the several States, subject only to the dormant control of the legislature of the State in which they were situated, and from which they derived their corporate powers. Neither the dominant feeling of the country in favor of individual liberty, or the multiplication of licensing bodies, tended to arrest the gradual lowering of the terms upon which the doctorate in medicine could be obtained, and "the fact," says Dr. Rosa, "that the degrees conferred by the colleges became practically recognized throughout the whole country as a sufficient license to practice medicine in all its branches," gave the student an opportunity of obtaining a degree wherever it was granted upon the most convenient or easiest terms; fully justifying the complaint of the president of the Medical Society of New York, in speaking of medical colleges, to the effect that "the present necessary laxity in admissions and in final examination, fairly overwhelms the land with physicians, many of whom are only so by title." This is but describing a state of things that existed in our own country at no pre-historic time, against which great effort had to be made before it was brought within control, and practically to an end. Its bearing upon our subject is important in so far only as it no doubt influenced the institution and constitution of dental colleges in America, created in 1840 and afterwards.

The distinguished president of Harvard University, Dr. Eliot, in his admirable address on "Dental Education," divided the subjects which constitute the fitting education of the dental surgeon into those which are peculiar to the general and to the special surgeon, and those which are common to both. The general he estimates as three-fifths, and the special subjects as constituting two-fifths of the whole education; and there will be few dissentients to this division. Keeping Dr. Eliot's estimate in mind, but that the medical degree was given and taken on such easy terms, it would have been difficult from our standpoint to understand how it was that so many of the dental colleges from the first undertook to educate their students in medicine and surgery, in the presence of schools devoted to these subjects, furnished with all the multitudinous appliances necessary for success in teaching, and with teachers of experience and distinction; and with the further evil of withdrawing the dental student from advantageous association with the general student in the study of subjects common to general and dental surgery. The separation of the students by this limitation to a special school, engendered a distinction of social position to the obvious disadvantage of the dental practitioner, whose pretention to the necessary amount of medical or surgical knowledge would be challenged by those who had studied under more favorable circumstances and under the guidance of established teachers. For however little professional education may be forced upon the individual student, there has never been a time when a diligent and determined student could not acquire a competent knowledge of his profession in the medical schools of America or of our own country.

Even a casual study of the organization of the American dental colleges leads to the inevitable impression that the Americans were, and indeed still are, strongly and rightly impressed with the absolute need of thorough training; and although not in a position to enforce the acceptance, yet feel bound to offer to students every inducement to acquire a sound knowledge of the special subjects, and of the requisite manipulative skill. The general subjects appear to have received less attention, or, at all events, occupied in the college prospectus a less prominent position. In some cases, indeed, it would almost seem that a college faculty thought that a sufficient knowledge of general surgery could be acquired in the study of the special subjects of dental surgery.

It was, however, felt in this country that the prevailing medical education was, as a question of degrees, not in excess of what should be required of the dentist; but that while it fell short to the extent of two-fifths of the whole in the direction of strictly dental knowledge, it exceeded by two-fifths in special medical knowledge, the amount which could be reasonably asked of well-educated dental practitioners. This opinion was fully expressed in a memorial, addressed to the English College of Surgeons, in the following terms: "The memorialists do not suggest an education and examination *inferior* to that required of the medical practitioner; but propose a certain difference in *kind* only, not a difference in *degree*—an education and examination specially adapted to the requirements of the dental surgeon, as distinguished from that fitted for the general surgeon."

The value of the foregoing paragraph has not been fully or rightly estimated either here or elsewhere. Equality of education, professional or other, does not necessitate identity. A parson, a lawyer, and a doctor, may be equally well educated. The degree of education may be the same in all, but some of the subjects embraced in each profession will be different. So it may be with a dentist and a doctor; the degree of culture may be equal, but in part the subjects of study will be different.

It has been urged that dental should come as supplemental to medical knowledge, that the practitioner should be a medical man first and a dentist afterwards. This opinion might be sustained if the position were reversed—a dentist first and a doctor afterwards—provided all students, or even the majority, were sufficiently rich in money and time to extend the educational period from four to six years, or from three to four and a half, a condition of things which obtains neither here, nor, according to Dr. Eliot, in America.

Four years are allotted to the study of medicine, and the medical student has not an hour to spare for any other subject, hence it becomes needful to determine what subjects in the medical curriculum can be lessened in extent or wholly omitted, so as to find time within the same four years for the effective study of dental surgery as a science and practice. This problem has not, perhaps, been wholly solved, but as the latest organizers of a complete scheme of compulsory dental education, it is hoped we may claim to have provided the most complete curriculum hitherto brought into a national use. The details of this were determined by a committee of the Medical Council, consisting of the representatives thereon of the medical authorities which, under the Dentists Act, grant dental qualifications, the twenty years' experience of the College of Surgeons of England being placed at their disposal. They reported in favor of it, and the Council adopted without material variation, the curriculum originated by the aforesaid College. The unconditional insistance upon an attested preliminary education before a person is allowed to commence his professional studies, is a feature of great importance in the existing regulations, inasmuch as it insures to the student an amount of knowledge and of mental training which renders him competent to understand without difficulty the language of science, and to follow with comparative ease the methods of scientific instruction and investigation. Before this great educational step was taken, pupils not uncommonly entered upon their professional studies so poorly informed that much time was lost in the attendance upon lectures which they but very imperfectly understood, and consequently, at the outset several lectures had to be delivered for the purpose of general instruction, and thus of preparing the student to take advantage of the subsequent course, rather than of imparting available medical knowledge. Much has been said against the vast number of lectures students have been required to attend, and especially against repetitions, and the objection is no doubt valid now that preliminary education is enforced, and the students thereby enabled to learn as much from one course as they formerly did from two courses. There may be difference of practice, but there can be no difference of opinion here or elsewhere as to the advantage to the student of an attested preliminary education.

Before entering upon the consideration of the instruction common to a medical and dental education, I will quote a few sentences from an "Address on the Study of Physiology," by Dr. T. M. Purser, and ask you to read therein Dental Surgery for Medicine:—

"I have said your business here is to learn medicine; and you learn the other subjects only as stepping stones to this; you do not come here to be made anatomists, or chemists, or physiologists. If you want to be an anatomist you must give your life to it; and so of the other sciences. But you learn those parts of these sciences which are essential, in order that you may take the next step safely; so much anatomy, physics, and chemistry, as are essential for physiology; and so much physiology as is essential for medicine, of which you should know all that is known."*

I will venture also to bring to your notice the following relevant paragraphs from the address of Dr. Michael Foster, in which he contends that topographical anatomy, which has hitherto been studied in some part as a mere mental training, should now to a certain extent give way in favor of a more complete knowledge of physiology. He says: "The details of topographical anatomy have the peculiar feature that, although they can only be learned with infinite pains and labor, unlike other things hard to learn. they vanish and flee away with the greatest ease. I would confidently appeal to any audience of practical men, how much of the huge mass of minute facts, which,

*British Medical Journal, November 13, 1880.

in their youth they gathered with so much toil, remained fresh in their minds two years after they passed the portals of the college; how much now remains to them beyond a general view of the parts of the human frame, and a somewhat more special knowledge of particular regions, their acquaintance with which has been maintained by more or less frequent operations. I would confidently ask them what is the ratio, in terms of money or any other value, which the time spent in those early anatomical struggles—say over the details of the forearm—bears to the amount of that knowledge remaining after twenty, or ten, or even five years of active practice, or to the actual use to which that knowledge has been put."

Dr. Burdon-Sanderson, in his introductory lecture, says:—"The precious years which immediately precede a man's entry on professional duty, are far too valuable to be wasted in learning anything he does not intend to retain."*

If we keep in mind these lately expressed and published opinions of these distinguished teachers, we shall be qualified to form a just estimate of the value of the current dental curriculum, regarded as a training in the principles of medicine, and of its relations to the current medical curriculum. Without substantial difference there are some slight variations in the divisions, and even in the designations of the lectures required by the several surgical colleges. On this account it will be convenient in making a comparison to take the respective courses of study of the English college; and the more so as its dental curriculum has been in successful operation for the best part of twenty years.

If, then, we refer to the tabulated statement given at p. 293 of the June number of this Journal, it will be seen that the dental student is required to attend one winter (six months') course of lectures on anatomy in a recognized medical school; and a second like course, or in lieu thereof a course on the head and neck. He is required to have dissected for nine months, in other words, during a winter and a summer session. The medical student on the other hand must attend with no alternative two winter sessions of anatomical lectures, and dissect during two winter

*British Medical Journal, October 9, 1880.

sessions, or twelve months. In fact, the dental student is relieved of part of one course of lectures, and of three months' dissection. But if any credit is to be given to the opinions I have quoted, enough, surely, remains for the education even of the medical, and certainly of the dental student. We know quite well, the knowledge of a subject got up merely for the purposes of a pass will not be retained, and who will contend that a minute knowledge of the anatomy of the foot will be of sufficient practical worth to the dentist to be retained in his memory, and if not to be retained, then precious time, Dr. Burdon-Sanderson says, should not be wasted in its acquisition. That which is true of the foot, is true also of the minute anatomy of many other parts of the body, with the treatment of which in disease the dental surgeon is not either directly or indirectly concerned.

A winter six months' course of lectures on physiology is required alike of each, but the dental student is excused the thirty lectures, or meetings of the class, on practical physiology, which are compulsory on the medical student. He will do well to decline this exemption; for a full knowledge of physiology is equally required for the intelligent practice of any and each branch of surgery. It is of all subjects the most interesting, and time can not be misspent by student in any manner of its study; neither need we fear that the knowledge of physiology will be lost either to ourselves or those who may ask our services. The attendance upon one course of lectures upon surgery during one winter session is required in each curriculum, but the attendance upon a six months' course of practical surgery is not required of the dental student.

A course of lectures upon chemistry and a three months' course upon practical chemistry is required in each curriculum, and in like manner a course upon materia medica, and also upon the practice of medicine.

A course of lectures upon forensic medicine, midwifery, pathology, practical pharmacy, and vaccination are replaced by other subjects in the dental curriculum.

We now come to attendance upon the practice of a general hospital. The medical student attends the surgical practice during three winter and two summer sessions, while the dental student attends during two winter sessions. The former is required to attend clinical lectures on surgery for two winter and two summer courses, but two winter courses only are required of the latter. And here the pupilage of the dental student at a medical school and general hospital ends. He is not required to attend the six months' dressership, the post mortem demonstrations, the practice of medicine and clinical medicine of the medical curriculum. But omitting these, can it be said with any show of truth, that the dental student has not had ample opportunities of acquiring a sound knowledge of the principles and practice of surgery? And if in the individual case that knowledge has not been acquired, it will be for the surgical section of the Board of Examiners to refuse the qualification of which they, with the dental section, are the constituted guardians of the public interest.

The all important special subjects compressed in the dental curriculum now claim our attention. In these the medical student takes no part, while it is by the exercise of them, under the direction of his general medical knowledge, that the dentist takes and holds his place in society.

The conditions imposed upon dental students are, that he shall, subsequent to his having passed the preliminary examination in general knowledge common to the dental and medical student, have devoted four years to the acquirement of professional knowledge; have been engaged during a term of three years in the acquirement of a practical knowledge of clinical dentistry under a competent instructor; have attended and taken part in the dental practice of a recognized dental hospital, or the dental department of a recognized general hospital, during a period of two years; have attended two courses, or not less than twenty-four lectures on dental anatomy and physiology, human and comparative; two courses, or not less than twenty lectures on dental surgery, and not less than twelve lectures on metallurgy, and a like course on mechanical dentistry.

These then are the subjects and conditions which take the place of those remitted from the medical curriculum, and who can justly say they do not impose a tax equal to the remission, upon the intelligence, the industry, and the time of the student? It may indeed be contended that a greater load is imposed, for it is the opinion of those engaged in instruction, and of those recently instructed, that nothing can be remitted from the special division of the curriculum. The hospital attendance must be exacted almost day by day during the two specified years, in order to attain adequate manipulative skill, without which the practitioner would be as the musician who can not play, the artist who can not draw, the sculptor who can not use the modeling tool or the chisel, or the dental critic who should be able to surpass but can not equal, the work he condemns in others. It is one thing to know the scientific principles of the art, but it is quite another to carry them into effect. This requires an amount of manipulative power, which can only be attained by long and careful practice under a competent instructor. The fingers must become unconsciously obedient to the will, they must follow it automatically as the fingers of the skilled pianofortist execute the mental reading of the work he is playing, or as the hand of the sculptor produces the form the mind has conceived. Short of this unbidden obedience of hand, the performer would be but an amateur, and his professional life one long apology.

It will be admitted by all that skill of hand can be attained only by long practice, and few will contend that one time is as good as another for the training. Mr. Fawcett has told us that the blind may be taught a bread-winning trade in their youth, but that adults who have lost their sight can not acquire sufficient skill to secure independence. We know that successful musicians and artists commence their studies in youth, and have given promise of power before they have attained to manhood. If we turn to the artisan class it will be found that he who fails to acquire skill of hand during his apprenticeship, seldom attains to excellence afterwards. There is no reasonable ground for doubting that the hand in youth develops anatomically in the direction of its exercise, and acquires thereby a power in that exercise to which the adult hand seldom attains. These facts have an important bearing upon the question of the time at which the dental student should proceed with his practical education, for the skill needed by the dentist is inferior to none of these. The results of professional examinations fully establish the fact that the medical and dental curriculum can not be honestly fulfilled in the same four years. Yet it has been said that the practitioner should be a surgeon first, and a dentist afterwards, or in other words, the entrance upon the special division of the dental curriculum should be delayed until the surgical education is completed, thus deferring the manipulative training to a period when the attainment of excellence is difficult, and in its highest degree, perhaps impossible. To devote the days of our youth to the acquisition of knowledge we do not intend to increase, to the exclusion of the knowledge by the exercise of which we propose to gain our bread, would be, I contend, a great error, and the more so, as the remitted portions of the medical curriculum can, if desired, be taken up when the dental education is completed.

My strong advocacy of special must not be interpreted as indifference to medical qualifications. I would give every possible encouragement to the attainment of the latter, not, however, as a substitute for, but as a supplement to, the dental degree. Educationally, the relations of the membership to the dental license may be regarded in the same light as the relation of the fellowship to the membership is regarded. This view will indeed take effect in certain appointments. In many of our hospitals, although the membership of the College of Surgeons is a full qualification, the governing body require that their surgical officers shall be Fellows of the College. Whenever the Fellowship of his College is required of a candidate—provided the Fellowship implies a higher degree of professional knowledge than the Membership—it may justly be required of the dental candidate for office that he shall possess the Membership in addition to the dental license of his College.

The profession at large must be congratulated upon the recent determination to enter in the Dentists' Register surgical degrees as additional qualifications. It may be said that this should have been done from the first, but those who have practical experience in bringing an Act into full operation know quite well that success requires patience, perseverance, and last, but not least, forbearance.

Upon the question of examinations and examiners I need say little. The former will, in their character, follow the lead of medical examinations, and it is provided in the Dentists' Act that should the conjoint scheme come into operation in medical, it will do so, also, in dental examinations.

The examiners are the guardians, on the part of the public, against incompetence, and should, as a matter of course, be independent of the pecuniary success of the schools, and collectively irresponsible for the professional instruction of the persons they are called upon to examine.

In reviewing the task imposed on the student, it may be asked whether I have not overstated the amount of special training needed to ensure the acquisition of the necessary manipulative power. I would answer, No, with all the emphasis of which I am capable. For I contend that a high degree of skill of hand is absolutely necessary to professional competence—that competence is necessary to self-respect—and that self-respect is necessary to that professional rectitude, without which personal comfort in practice would be imperilled, and professional status would be but a shallow fiction.

Furthermore, that with the existing opportunities a high degree of skill can be gained by perseverence and due expenditure of time in pupilage, and that it is the bounden duty of the teacher to press, and for an examiner to demand, its possession.

Such, then, are the lines upon which the study of dental surgery have been drawn—so drawn as to secure adequate knowledge and skill in the practitioner—and with the view, therefore, to a certain difference in *kind*, but to equality in *degree*, between the compulsory education of the medical and of the dental practitioner.

If in this imperfect sketch I have entered at certain points too far into detail, or occupied too much time in their description, extenuation for my proclivity may be pleaded on the score of the high degree of satisfaction, not to say pardonable pride, which the surviving members of my generation feel in seeing an educational scheme, in the origination of which they took part, completed, and rendered national, and a calling heretofore of undefined position elevated by the legislature to the rank of a learned profession.

